# Atlas of lysine propionylation of human right atrium and ALDH6A1-NADH pathway in new-onset atrial fibrillation after coronary surgery

**DOI:** 10.1038/s42003-025-08264-9

**Published:** 2025-06-04

**Authors:** Yi Zhang, Huan-Xin Chen, Hai-Tao Hou, Ying-Qi Liu, Zhuo Chen, Qin Yang, Guo-Wei He

**Affiliations:** 1 Department of Cardiovascular Surgery & The Institute of Cardiovascular Diseases, TEDA International Cardiovascular Hospital, Tianjin University & Chinese Academy of Medical Sciences, Tianjin, China; 2 Tianjin Key Laboratory of Molecular Regulation of Cardiovascular Diseases and Translational Medicine, Tianjin, China; 3 Division of Cardiothoracic Surgery, Department of Surgery, Oregon Health & Science University, Portland, OR USA

**Keywords:** Atrial fibrillation, Translational research

## Abstract

Lysine propionylation modification (Kpr) plays an important role in the pathogenesis of several cardiovascular diseases, but the role of Kpr in postoperative atrial fibrillation (POAF) is unclear. Here, we established an atlas of proteomics and propionylation proteomics in the atrial appendage tissues from 28 CABG patients, exploring the role of Kpr proteins in the occurrence of POAF. The Kpr of ALDH6A1 was most significantly increased on Lys113 (2.25 folds). The activity of ALDH6A1 increased due to higher binding energy of propionylated ALDH6A1 and NAD^+^, causing an increase in NADH levels in cells and triggering abnormal energy metabolism. Furthermore, the increase in NADH levels triggered the accumulation of reactive oxygen species, which may cause oxidative stress, resulting in the development of AF. This study reveals the important role of ALDH6A1-NADH pathway in POAF, and provides new insights for exploring the pathogenesis of POAF in CABG.

## Abstract (web summary)

The alterations of lysine propionylation modifications in the human right atrium reveal the important mechanism of ALDH6A1-NADH pathway in the pathogenesis of new-onset atrial fibrillation following coronary artery bypass grafting.

## Introduction

Coronary artery disease is the most common type of cardiovascular diseases^1^. New-onset postoperative atrial fibrillation (POAF) is a common complication after coronary artery bypass grafting (CABG) in patients with coronary artery disease^2^. The incidence of POAF is reported to be about 20–50%^3–5^. POAF not only increases the risk of long-term atrial fibrillation (AF) in patients, but also leads to stroke, heart failure, and even death, seriously affecting the health of patients and the effect of surgical treatment of coronary artery disease^6–8^. The occurrence of AF is affected by many factors, including age, gender, race, genetics, and a variety of other comorbidities such as hypertension, diabetes, heart failure, coronary artery disease, valvular disease, and obstructive sleep apnea-hypopnea syndrome^9–11^. Studies have shown that the pathogenesis of AF is related to atrial electrical remodeling, structural remodeling, metabolic regulation, oxidative stress and dysregulation of ion channels such as sodium, potassium, and calcium channels^12–19^. In particular, aging is the major risk factor for the occurrence of POAF^1,3,4^. In fact, the age for the POAF patients was 65.2 years old vs. 61.8 years old in patients with normal heart rhythm in a large cohort of patients in a recent report^4^. Nevertheless, the specific pathogenesis of POAF is still unclear.

Protein post-translational modifications (PTMs) are chemical changes on the amino acid side chains of proteins through covalent modification or proteolysis^20,21^. PTMs are important regulatory mechanisms in organisms, which affects the activity, function, and stability of proteins by changing their structure, and plays an important role in cardiovascular diseases^22–24^. With the development of omics technology, more and more novel lysine acylation modifications have been discovered, including succinylation, crotonylation, propionylation, butyrylation, malonylation, glutarylation, 2-hydroxyisobutyrylation, and lactylation, which provide new evidence for the prevention, diagnosis, and treatment of cardiovascular disease^25–30^.

Lysine propionylation (Kpr) is a PTM that was first discovered in histones in 2007^31^. Propionyl-CoA is a key molecule in amino acid and odd-chain fatty acid catabolism, and it is the donor of Kpr^32^. Kpr has been found to exist in both histones and non-histone proteins^33^. Histone propionylation is a marker of active chromatin and provides novel epigenetic regulation for cell metabolism^33,34^. In a MOZ-TIF2-driven mouse model of leukemia, the level of histone H3 propionylation at lysine 23 (H3K23pr) was elevated and closely associated with the regulation of gene transcriptional activity^35^. Propionylation of K198 and K203 of phosphate-sensing regulator (PhoP) reduced the transcriptional activity of PhoP in *Saccharopolyspora erythraea*^36^. A small-sample study revealed that the propionylation at lysine 17 of histone H2B (H2BK17pr) regulated proteostasis^37^. An analysis of Kpr in the intestinal samples of zebrafish showed that superoxide dismutase 2 (Sod2) was propionylated at K132, which was related to oxidative stress^38^.

There are few studies on propionylation modifications in cardiovascular disease^39^. Branched-chain amino acids (BCAAs) metabolism is a major source of propionyl-CoA, participating in Kpr to regulate gene expression in cardiovascular diseases^40,41^. Histone PTMs are important in epigenetics and play an important role in regulating gene transcriptional activity^42^. A recent study^43^ showed that reducing the concentration of BCAAs may reduce collagen gene expression and fibroblast proliferation in cardiac hypertrophy by modulating cellular epigenetics. Therefore, reducing BCAAs intake improves cardiac stress response in mice by decreasing H3K23pr level in the heart, thereby alleviating cardiac hypertrophy and heart failure^43^. Further, it was reported^44^ that BCAAs catabolism was an important regulator of platelets activation and was related to arterial thrombosis. The specific mechanism was to enhance the K255 propionylation of tropomodulin-3, thereby promoting platelet activation and thrombosis^44^. However, the role of propionylation in AF (especially POAF) has not been reported yet.

In this study, we constructed the proteomics and propionylation proteomics atlas of right atrial appendage tissues discarded during CABG surgery in both patients who later remained in sinus rhythm (SR) or developed POAF after CABG, and explored the correlation and mechanism by comparing the Kpr protein between the patients who developed POAF and postoperative sinus rhythm (POSR) after CABG.

## Results

### Association atlas of proteomics and propionylation proteomics in the right atrial appendage tissues

**Fig. 1:**
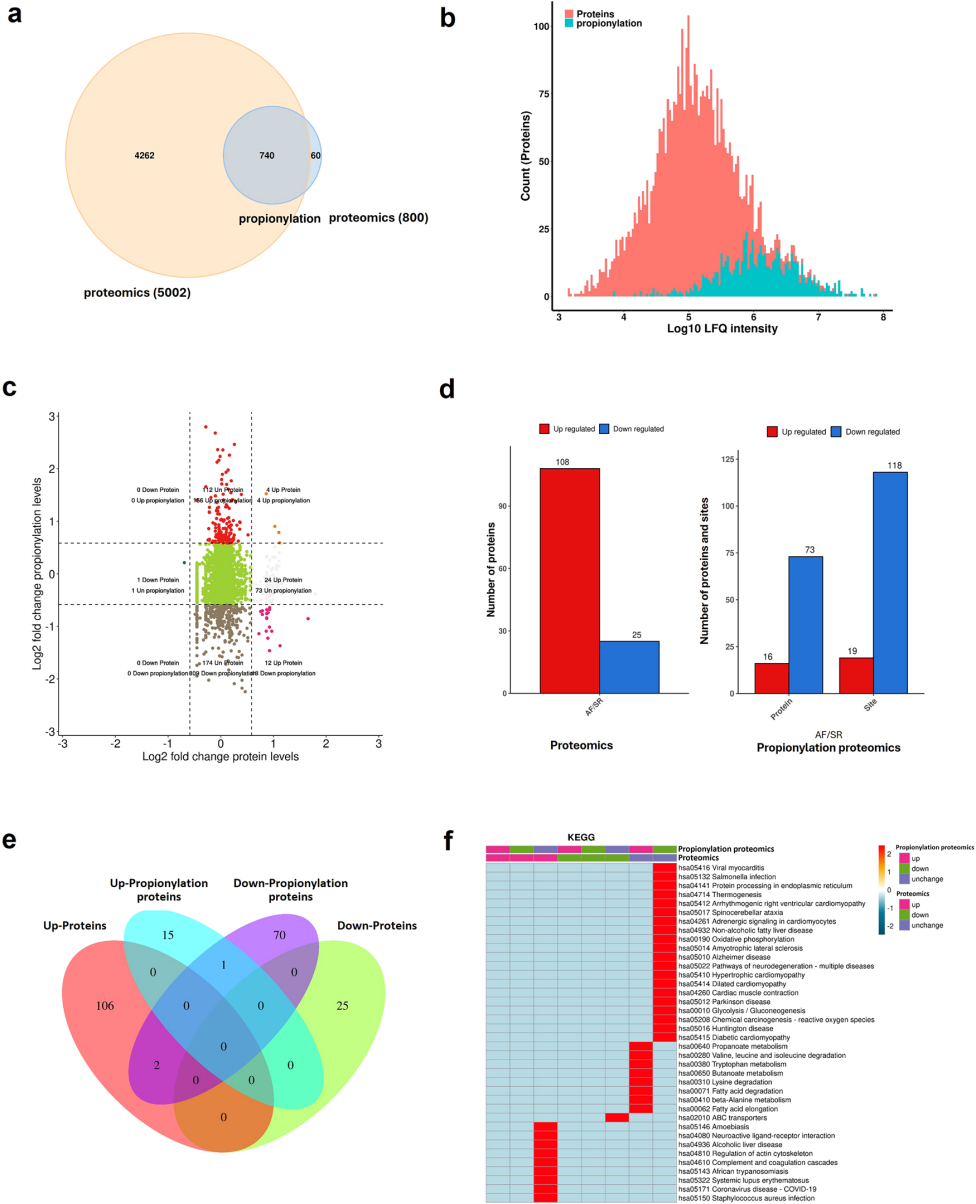
Association atlas of proteomics and propionylation proteomics in intraoperative right atrial appendage tissues of patients with POSR and POAF. **a** The intersection of all proteins identified by the two omics (n = 5 per group). **b** Distribution of all protein abundance quantified by the two omics. **c** Nine-quadrant diagram of comparative analysis of differentially expressed proteins between the two omics. Different colors represented different expression patterns. **d** Distribution statistics of differential proteins and differential sites. The left side was the proteomics results, and the right side was the propionylation proteomics results. **e** Venn diagram for comparative analysis of differentially expressed proteins between the two omics. The numbers in the figure represented the number of proteins contained in the intersection. **f** Heat map of functional enrichment differences between the two omics of differently classified proteins on KEGG pathway. The redder the color, the more significant the enrichment.

The discarded right atrial appendage tissues were collected during surgery for quantitative proteomics and propionylation proteomics analysis using the label-free 4D method, and the results of the two omics were analyzed by correlation (Fig. 1, Fig. S1). All patients selected for this study were in SR before CABG. After CABG, the patients were allocated into the POSR group (SR, n = 5) or into POAF group (AF, n = 5), depending on their heart rhythm.

A total of 5002 proteins were identified in the proteomics and 800 proteins were identified in the propionylation proteomics, of which 740 proteins were overlapped (Fig. 1a). The histogram showed the distribution of the intensity values of all quantified proteins in the proteomics and propionylation proteomics (Fig. 1b). There were 108 up-regulated proteins and 25 down-regulated proteins in the proteomics. In the propionylation proteomics, 16 proteins were up-regulated and 73 proteins were down-regulated. The nine-quadrant diagram, bar chart and venn diagram all showed the distribution of all differentially expressed proteins in the two omics (Fig. 1c–e). In addition, we used heat maps to visualize the connections between different classified proteins in the two omics in terms of GO functions (Fig. S1a–c), KEGG pathways (Fig. 1f) and domain binding (Fig. S1d). Detailed data of the proteomics results are shown in Fig. S2.

### Analysis of lysine-propionylated proteins

**Fig. 2:**
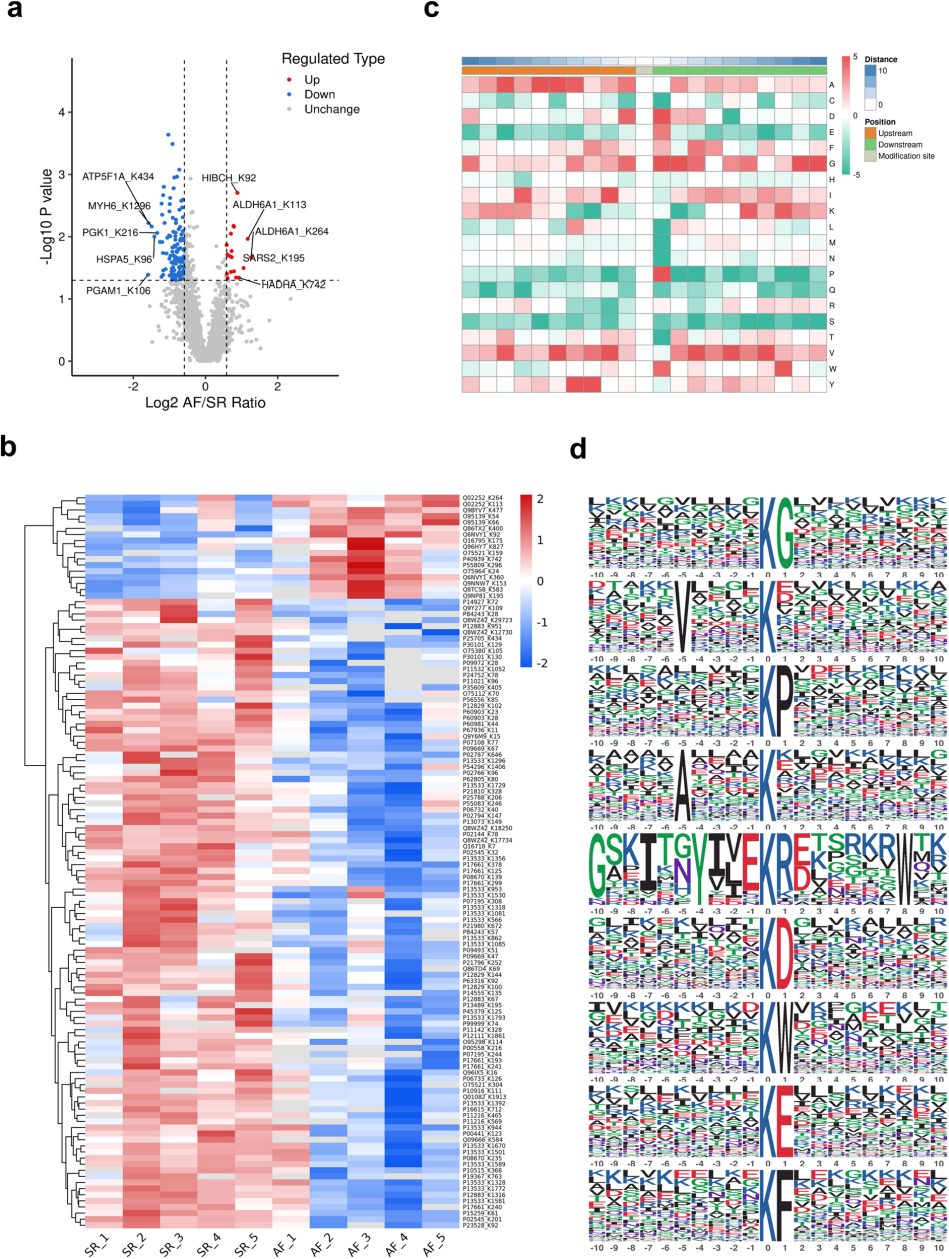
Proteomics analysis of Kpr in intraoperative right atrial appendage tissues from patients with POSR and POAF. **a** The volcano plot of differential Kpr sites. The red points indicated significant up-regulation, and the blue points indicated significant down-regulation. The information on the differentially modified sites for Top5 up- and down-regulation were also marked in the figure, respectively. **b** The heat map of differential Kpr sites. Red represented high expression and blue represented low expression. **c** Motify analysis heat map of 10 amino acids upstream and downstream of the modification sites. Yellow or green indicated upstream or downstream, respectively. **d** The diagram of motif logo showed the flanking sequence preferences for all Kpr sites.

The propionylation proteomics data were normalized based on the proteomics data of the same sample, thus eliminating the effect of protein expression on modification level. There were 3018 modification sites on 800 proteins identified, of which 2129 modification sites on 557 proteins were available for quantitative comparison. Compared with the SR group, 19 Kpr sites of 16 proteins were significantly up-regulated (AF/SR > 1.5, *P* < 0.05) and 118 Kpr sites of 73 proteins were significantly down-regulated (AF/SR < 1/1.5, *P* < 0.05) in the AF group. The volcano plot visualized the distribution of each differential Kpr sites between the two groups, where the top 5 differentially expressed Kpr sites in up- and down-regulation were labeled (Fig. 2a). The heatmap demonstrated the relative expression of all differential Kpr sites in different samples, with red color representing high expression and blue color representing low expression (Fig. 2b). Peptide sequences of 10 amino acids up-stream and down-stream of all Kpr sites were identified using MoMo analysis based on the motify-x algorithm (−10 to +10), and the frequency of occurrence of 20 amino acids near the Kpr sites was presented in the form of a heat map (Fig. 2c). In addition, a total of 9 Kpr motifs were identified in 1677 modified peptides (Fig. 2d).

### Bioinformatics analysis of differentially Kpr proteins

**Fig. 3:**
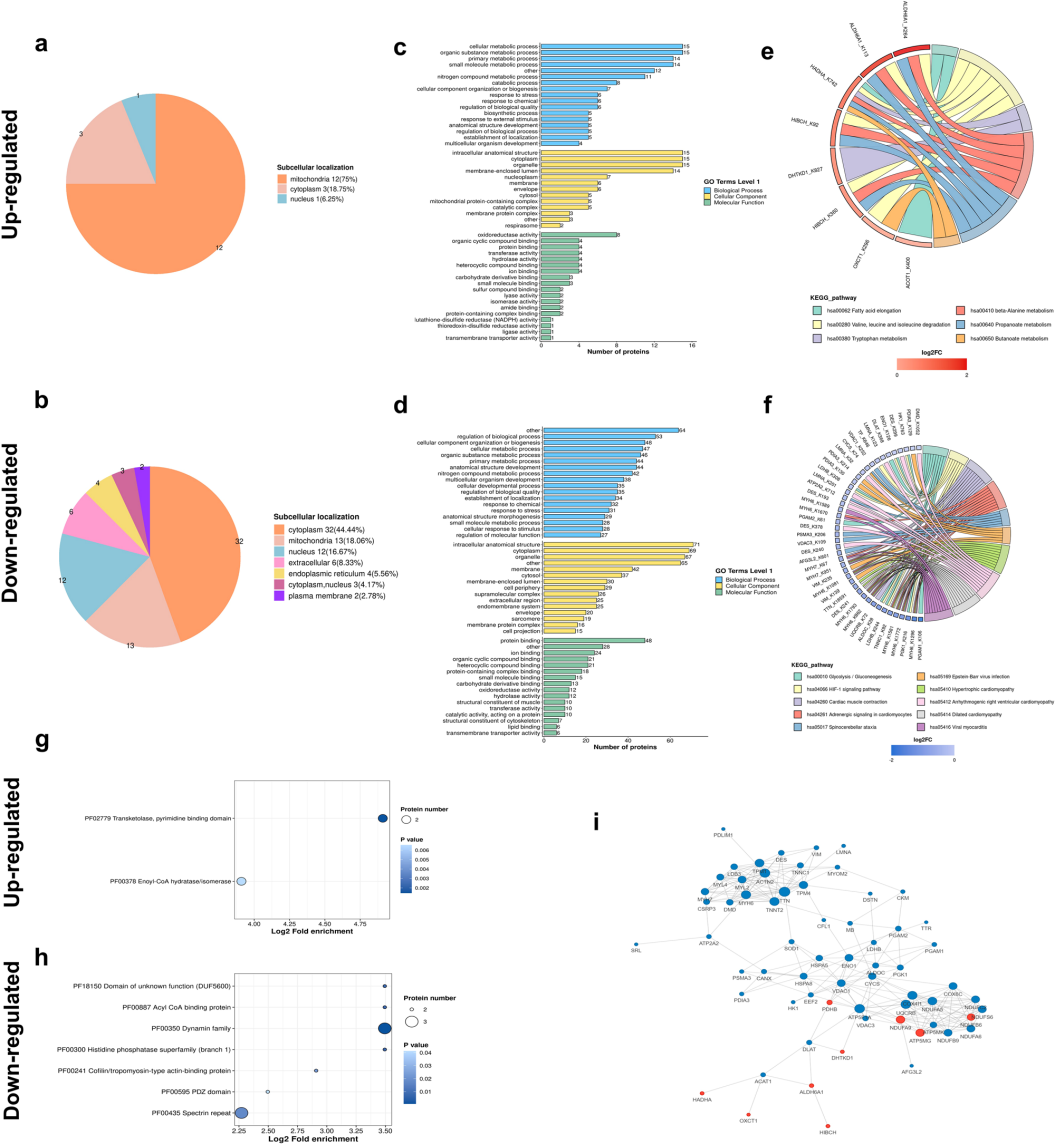
Functional classification and functional enrichment analysis of differentially Kpr proteins. The number of differentially Kpr up-regulated proteins (**a**) and differentially Kpr down-regulated proteins (**b**) in different subcellular structural types. GO enrichment analysis of differentially Kpr up-regulated proteins (**c**) and differentially Kpr down-regulated proteins (**d**) based on biological process, cellular component, and molecular function. KEGG pathway analysis of differentially Kpr up-regulated proteins (**e**) and differentially Kpr down-regulated proteins (**f**). Protein domain enrichment analysis of differentially Kpr up-regulated proteins (**g**) and differentially Kpr down-regulated proteins (**h**). **i** Diagram of the tightest 50 differential protein-protein interaction network. Blue represented down-regulated modified proteins and red represented up-regulated modified proteins.

We performed bioinformatics functional classification and functional enrichment analysis of the 16 up-regulated Kpr proteins and 73 down-regulated Kpr proteins (Fig. 3).

Functional classification included subcellular localization and GO classification. The results of subcellular localization showed that up-regulated Kpr proteins were mainly located in mitochondria (75%), cytoplasm (18.75%) and nucleus (6.25%) (Fig. 3a), and down-regulated Kpr proteins were mainly located in cytoplasm (44.44%), mitochondria (18.06%) and nucleus (16.67%) (Fig. 3b). The GO classification results showed that up-regulated Kpr proteins were mainly enriched for biological processes such as cellular metabolic process (93.8%), organic substance metabolic process (93.8%) and primary metabolic process (87.5%); cellular components such as intracellular anatomical structure (93.8%), cytoplasm (93.8%) and organelle (93.8%); and molecular functions such as oxidoreductase activity (50%), organic cyclic compound binding (25%), protein binding (25%) and transferase activity (25%) (Fig. 3c). Down-regulated Kpr proteins were mainly enriched for biological processes such as regulation of biological process (72.6%), cellular component organization or biogenesis (65.7%) and cellular metabolic process (64.4%); cellular components such as intracellular anatomical structure (97.2%), cytoplasm (94.5%) and organelle (91.8%); and molecular functions such as protein binding (65.8%), ion binding (38.4%) and organic cyclic compound binding (28.8%) (Fig. 3d).

Functional enrichment mainly included KEGG pathways and protein domains. KEGG pathway analysis indicated that differentially up-regulated Kpr proteins were predominantly enriched for beta-Alanine metabolism (hsa00410), propanoate metabolism (hsa00640), valine, leucine, and isoleucine degradation (hsa00280), fatty acid elongation (hsa00062), butanoate metabolism (hsa00650) and tryptophan metabolism (hsa00380) (Fig. 3e), and differentially down-regulated Kpr proteins were mainly enriched for glycolysis/gluconeogenesis (has00010), cardiac muscle contraction (hsa04260), hypertrophic cardiomyopathy (hsa05410) and so on (Fig. 3f). In addition, protein domain enrichment analysis revealed that differentially up-regulated Kpr proteins were enriched for transketolase, pyrimidine binding domain (PF02779) and Enoyl-CoA hydratase/isomerase (PF00378) (Fig. 3g), and down-regulated Kpr proteins were enriched dynamin family (PF00350) and spectrin repeat (PF00435) and others (Fig. 3h). The protein-protein interaction network demonstrated the interactions between all Kpr differential proteins (Fig. 3i).

### The Kpr Level of ALDH6A1 was up-regulated in the intraoperative right atrial appendage tissue of POAF patients

**Fig. 4:**
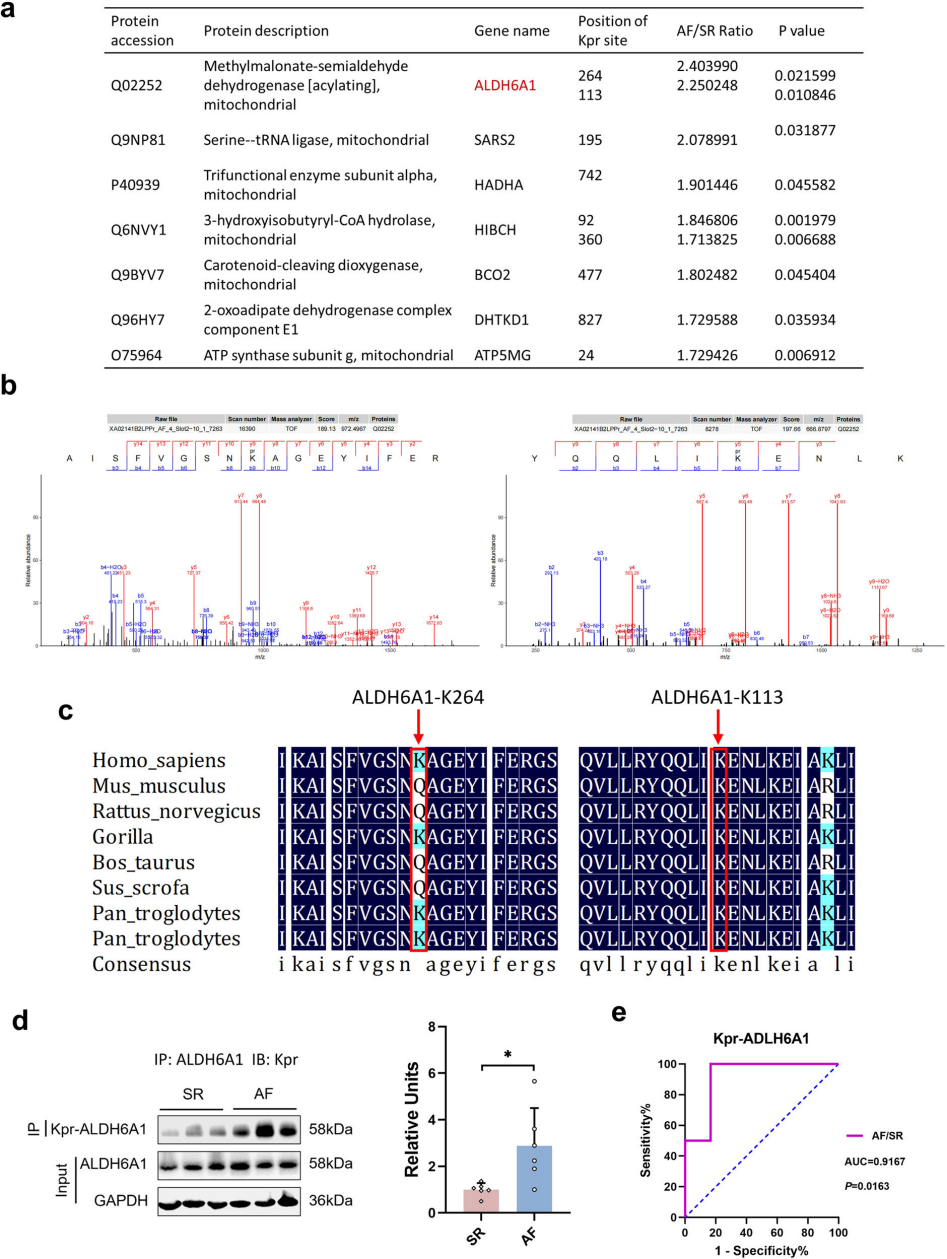
Up-regulated propionylated ALDH6A1 in right atrial appendage tissue of POAF. **a** Seven proteins with nine differential Kpr sites had significantly higher levels (fold change > 1.7, *P* < 0.05). **b** Mass spectrometry peak maps of the ALDH6A1 K264 and K113 propionylation. **c** Protein homology sequence analysis of the ALDH6A1 264 and 113 sites in different species. ALDH6A1 K264 and K113 were highlighted red. **d** Validation of ALDH6A1 propionylated expression in POSR and POAF right atrial appendage tissues by IP and WB (n = 6 per group). **e** ROC curve of Kpr-ALDH6A1 in the right atrial appendage tissues of patients with POSR and POAF (n = 6 per group). All experimental data were expressed as Mean ± SD. *P < 0.05.

Among all the up-regulated Kpr proteins and differential sites, 9 Kpr sites of 7 proteins had fold changes greater than 1.7, among which the 2 Kpr sites (K264 and K113) of aldehyde dehydrogenase family 6 member A1 (ALDH6A1) had the largest fold changes (Fig. 4a). ALDH6A1 was a methylmalonate-semialdehyde/malonate-semialdehyde dehydrogenase that catalyzed the irreversible oxidative decarboxylation of malondialdehyde and methylmalonic acid semialdehyde to acetyl-CoA and malonyl-CoA, and played an important role in lipid metabolism, amino acid metabolism, and oxidation reduction. ALDH6A1 is localized in mitochondria. In this study, it was mainly enriched in multiple KEGG pathways, such as valine, leucine, and isoleucine degradation (hsa00280), beta-Alanine metabolism (hsa00410), and propanoate metabolism (hsa00640) (Fig. 3e).

Compared with POSR patients after CABG, the propionylated levels of K264 and K113 in ALDH6A1 were increased in atrial appendage tissues of POAF patients by LC-MS/MS analysis (Fig. 4b). In addition, we compared the homology of ALDH6A1 264 and 113 sites in eight organisms, and found that all 113 sites were lysine conserved, while 264 sites were not, and then ALDH6A1 K113 was selected for subsequent validation experiments (Fig. 4c). Next, the Kpr level of ALDH6A1 was validated using IP and WB in the right atrial appendage tissues of new cohort patients with POSR (n = 6) and POAF (n = 6), respectively. The WB results demonstrated that the Kpr level of ALDH6A1 was significantly higher in the POAF group than that in POSR group, which was consistent with the results of propionylation proteomics (Figs. 4d and S3). In addition, we also demonstrated the relevance of Kpr-ALDH6A1 in distinguishing 6 POSR patients from 6 POAF controls by receiver operating characteristic (ROC) (AUC = 0.9167, *P* = 0.163, Fig. 4e).

### ALDH6A1-K113 propionylation increased the enzyme activity and intracellular NADH level

**Fig. 5:**
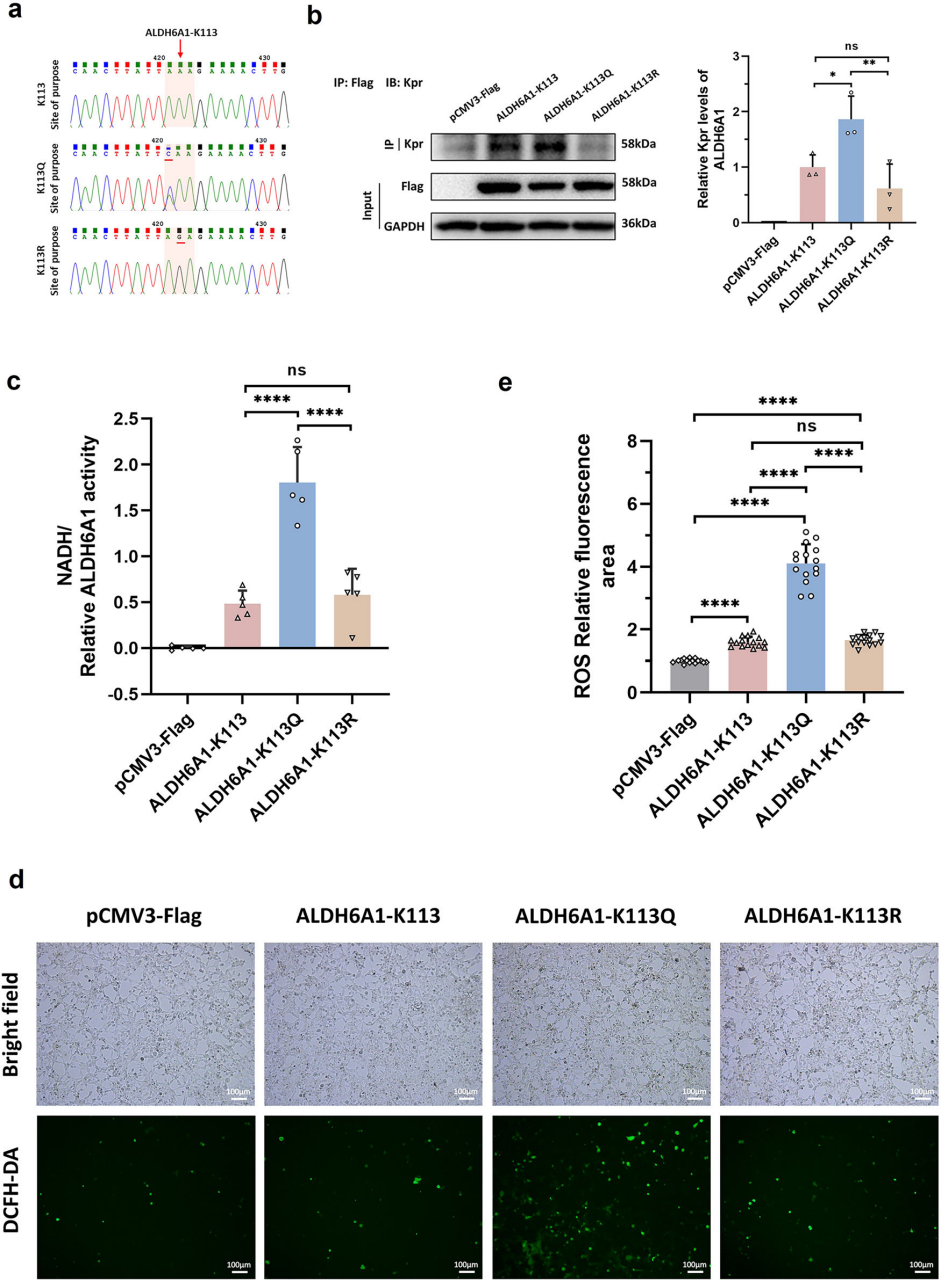
Kpr expression of ALDH6A1-K113, ALDH6A1-K113Q, and ALDH6A1-K113R in HEK-293 cells and cell function verification. **a** Sequencing chromatograms of pCMV3-ALDH6A1-K113, pCMV3-ALDH6A1-K113Q and pCMV3-ALDH6A1-K113R point mutation sites. **b** Kpr expression levels of over-expressed pCMV3-Flag, ALDH6A1-K113, ALDH6A1-K113Q, and ALDH6A1-K113R in HEK-293 cells were verified by IP. IP was performed with anti-Flag antibody, and then detected with Pan-Kpr antibody (n = 3 per group). **c** Detection of NADH levels in HEK-293 cells transfected with pCMV3-Flag, ALDH6A1-K113, ALDH6A1-K113Q, and ALDH6A1-K113R. ALDH6A1 levels were detected by anti-Flag antibody for normalization (n = 5 per group). **d** Bright field and fluorescence images of HEK-293 cells transfected with pCMV3-Flag, ALDH6A1-K113, ALDH6A1-K113Q, and ALDH6A1-K113R and incubated with DCFH-DA. Scale bars, 100 μm. **e** Statistical analysis of relative ROS positive areas in HEK293 cells (n = 15 per group). All experimental data were expressed as Mean ± SD. ns no significance; ****P < 0.0001.

In order to investigate the effect of ALDH6A1-K113 propionylation on enzyme activity and function, we purchased pCMV3-Flag (blank control) and pCMV3-ALDH6A1-Flag expression vectors (pCMV3-ALDH6A1-K113). The pCMV3-ALDH6A1-K113Q/K113R were obtained by point mutation to mimic the up- or down-regulation of Kpr level (Fig. 5a). The pCMV3-Flag and pCMV3-ALDH6A1-Flag (K113, K113Q, and K113R) plasmids were transfected into HEK293 cells, and the proteins were extracted for IP and WB validation after 36 h. The results showed that compared with the ALDH6A1-K113 group, the Kpr level in the ALDH6A1-K113Q group increased (Fig. 5b). In addition, the intracellular NADH level in the ALDH6A1-K113Q group was significantly higher than that in the ALDH6A1-K113 group and the ALDH6A1-K113R group (*P* < 0.0001, Fig. 5c). However, there were no significant differences in NADH level between the ALDH6A1-K113 group and the ALDH6A1-K113R group (Fig. 5c). NADH was a product generated by the catalytic reaction of ALDH6A1 that indirectly reflected the enzyme activity of ALDH6A1. The above experimental results indicated that ALDH6A1-K113 propionylation enhanced the enzyme activity of ALDH6A1 and increased the intracellular NADH level.

### ALDH6A1-K113 propionylation promoted accumulation of intracellular reactive oxygen species (ROS) by increasing NADH level

Previous studies have shown that increased intracellular NADH leads to the accumulation of ROS^17,45^. Therefore, we detected the intracellular ROS level in HEK293 cells after transfection with plasmids pCMV3-Flag and pCMV3-*ALDH6A1*-Flag (K113, K113Q, and K113R). The results showed that compared with the basic control group, over-expression of ALDH6A1 (K113, K113Q, and K113R) significantly enhanced the intracellular ROS green fluorescence intensity (Fig. 5d). Compared with over-expressed ALDH6A1-K113, the ROS green fluorescence intensity in ALDH6A1-K113Q cells increased significantly (*P* < 0.0001), while there was no significant difference in ALDH6A1-K113R (Fig. 5e). In addition, the intracellular ROS green fluorescence intensity of ALDH6A1-K113Q was significantly enhanced compared with ALDH6A1-K113R (*P* < 0.0001, Fig. 5e). These experimental results suggested that ALDH6A1-K113 propionylation may promote the accumulation of ROS by enhancing the intracellular NADH level.

### Increased binding energy between propionylated ALDH6A1-K113 and NAD^+^

**Fig. 6:**
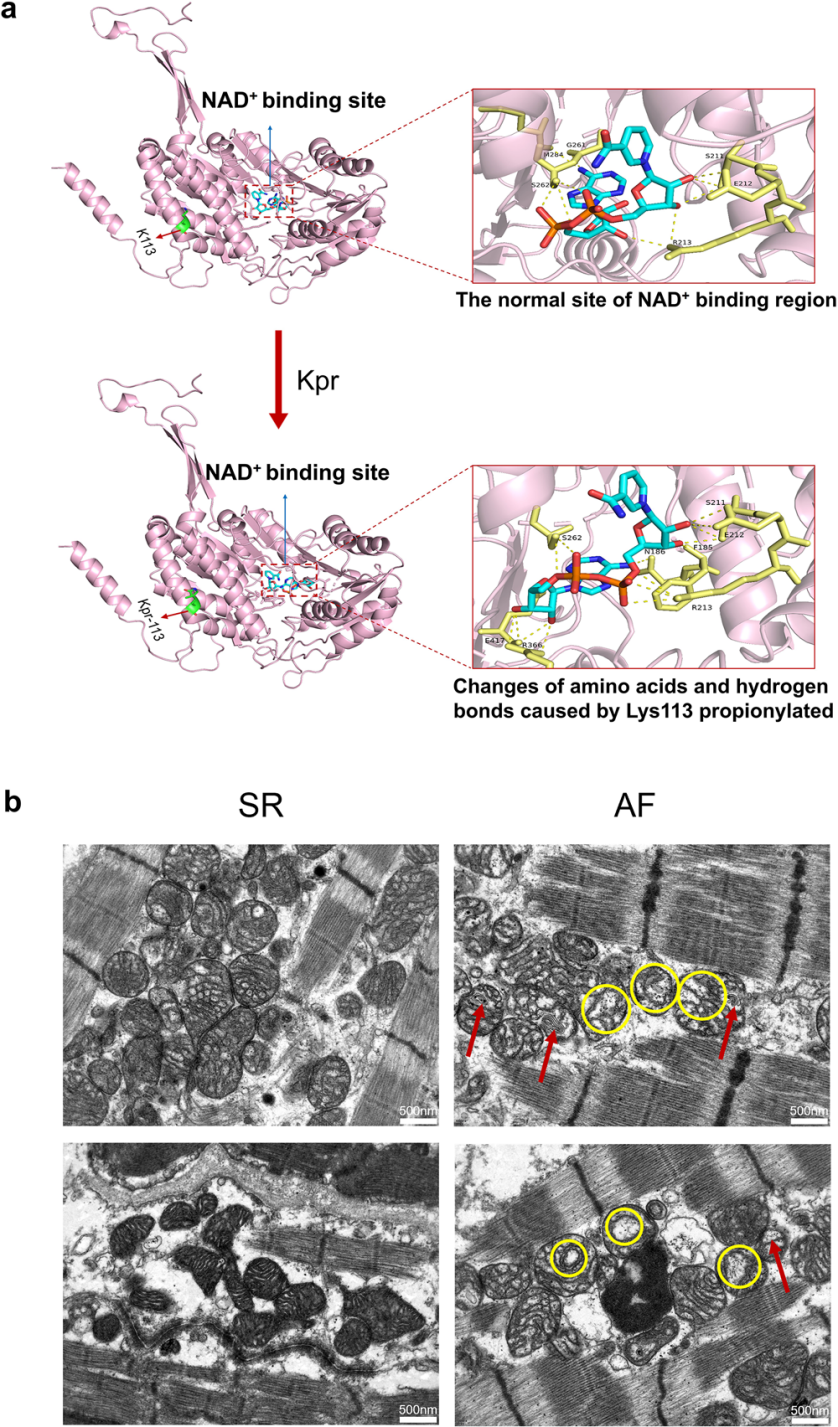
Molecular docking of ALDH6A1 with NAD^+^ and ultrastructural electron microscopic images of cardiomyocytes. **a** The binding conformation of ALDH6A1-NAD^+^ was simulated. The amino acids and hydrogen bonds in ALDH6A1 were changed by K113 propionylation, from 10 hydrogen bonds with 6 amino acids (S211, E212, R213, G261, S262 and M284) to 15 hydrogen bonds with 8 amino acids (F185, N186, S211, E212, R213, S262, R366 and E417). **b** Compared with the SR group, the ultrastructure of atrial cardiomyocytes in the AF group showed obvious mitochondrial swelling, vacuolization, and cristae damage. The yellow circles in the figure indicated mitochondrial swelling and vacuolization, and the red arrows indicated mitochondrial cristae damage (n = 3 per group).

Molecular docking technology was used to simulate the direct binding of ALDH6A1-K113 with NAD^+^ and ALDH6A1-K113 propionylation with NAD^+^. The docking results showed that the binding of ALDH6A1-K113 with NAD^+^ mainly formed 10 hydrogen bonds with 6 amino acids (Ser211, Glu212, Arg213, Gly261, Ser262, and Met284). After the K113 side chain of ALDH6A1 was modified by adding propionyl, the number and position of amino acids bound to NAD^+^ changed, and it became 15 hydrogen bonds with 8 amino acids (Phe185, Asn186, Ser211, Glu212, Arg213, Ser262, Arg366, and Glu417). The binding energy increased from −8.5 kcal/mol to −9.2 kcal/mol (Fig. 6a). Therefore, K113 propionylation enhanced the binding ability of ALDH6A1 with NAD^+^ and promoted the enzymatic activity of ALDH6A1.

### Ultrastructure of atrial myocytes before the major procedure of CABG surgery in patients with AF after CABG

Electron microscopic results showed that severe mitochondrial swelling, vacuolization, and mitochondrial cristae damage were observed in atrial myocytes of POAF patients before the major procedure of CABG surgery compared with those in the POSR control group (Fig. 6b).

## Discussion

In the present study, using quantitative proteomics and propionylated proteomics approaches, we found in the right atrial appendage tissues of patients with POAF compared to the patients without POAF after CABG that (1) the levels of Kpr proteins in the atrial appendage tissue of POAF patients were significantly changed, among which the Kpr level of ALDH6A1 at K264 and K113 were most significantly increased; (2) propionylated ALDH6A1 caused changes in cellular function and had significantly increased enzyme activity; (3) propionylated ALDH6A1 caused elevated intracellular NADH and ROS that are associated with oxidative stress and play an important role in the development of POAF; (4) the types of amino acids and the number of hydrogen bonds in the NAD^+^ binding region were altered and the binding energy was increased in propionylated ALDH6A1; (5) the ultrastructure of atrial myocytes in patients with POAF was altered before the major procedure of CABG, showing significant mitochondrial swelling and cristae damage.

A total of 89 differential proteins and 137 differential sites were identified by PTM proteomics study (Figs. 1d and 2a). Most of these up-regulated proteins were located in mitochondria, and they were enriched in multiple metabolic pathways (Fig. 3a, e). One of the proteins of greatest interest is ALDH6A1 that is the protein with the largest change in Kpr up-regulation at the K264 (2.40 folds) and K113 (2.25 folds) (Fig. 4a). However, the K264 site is not conserved and therefore it was excluded (Fig. 4c). In this study, we further validated the Kpr level of ALDH6A1 in the samples from a new cohort of the patients (Fig. 4d). The validation results on the Kpr level of ALDH6A1 were consistent with the omics study.

ALDH6A1, also known as Malonate and methylmalonate semialdehyde dehydrogenase (MMSDH), is a member of the aldehyde dehydrogenase (ALDH) family and is primarily found in the mitochondria of liver, kidney, and heart tissues^46^. It has been shown to be a biomarker for a variety of cancers^47–49^. The catabolism of BCAAs is one of the sources of ALDH6A1, which can use NAD^+^ as a coenzyme to oxidize aldehydes to generate NADH and propionyl-CoA, thereby regulating energy metabolism^50^. Studies have shown that ALDH6A1 is a key protein involved in lipid metabolism, immune inflammation, regulating redox levels, and various metabolic diseases^51,52^. Down-regulation of ALDH6A1 expression has been reported a biomarker of insulin resistance in skeletal muscle in type 2 diabetes mellitus^53^. Another proteomics study on the left ventricle in a rat model of epilepsy and found that the expression level of ALDH6A1 was significantly increased in epileptic myocardial tissues, providing a new target for mitigating myocardial injury^54^.

In this study, the generation of NADH in cells increased when ALDH6A1-K113 was mutated to ALDH6A1-K113Q, while there was no significant difference in the level of NADH when ALDH6A1-K113 was mutated to ALDH6A1-K113R (Fig. 5c). Since NADH was the product of the metabolic reaction in which ALDH6A1 participated in, these results also indirectly indicated that K113 propionylation increased the activity of ALDH6A1. Increased ALDH6A1 activity affected the metabolism of BCAAs and propionate, leading to an increase in downstream propionyl-CoA, which in turn changed protein propionylation levels and causing metabolic disorders. In addition, Kpr at the ALDH6A1-K113 site changed the conformation of the NAD^+^ binding region including the number of hydrogen bonds and amino acids, increasing the binding energy of ALDH6A1 with NAD^+^ (Fig. 6a).

**Fig. 7:**
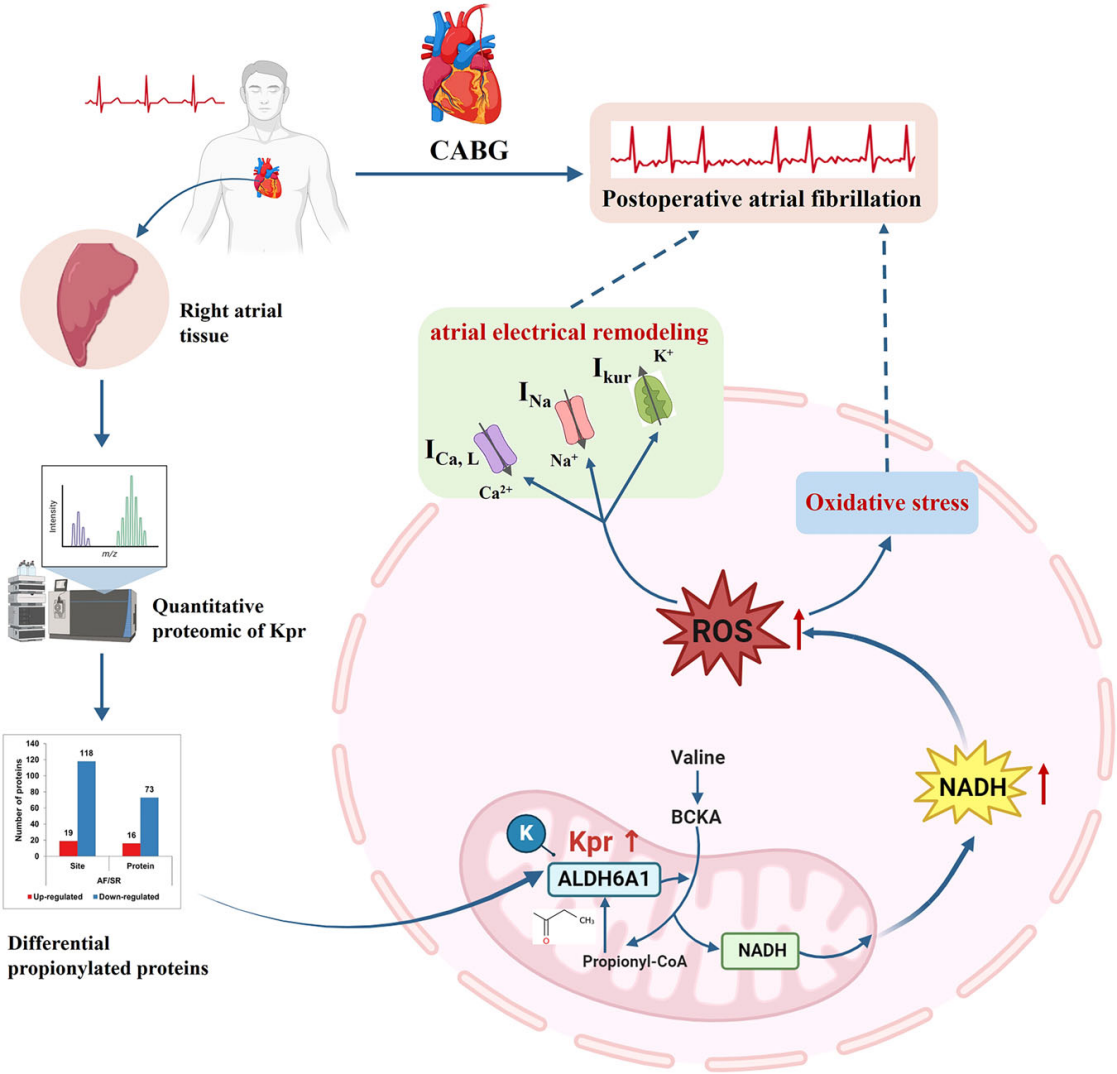
Schematic mechanism of intraoperative Kpr protein in patients with POAF after CABG. After CABG, patients with POAF increased the Kpr level of ALDH6A1 in right atrial appendage tissue and enhanced its enzyme activity, resulting in increased intracellular production of NADH and accumulation of ROS. The increase of intracellular ROS may cause oxidative stress and atrial electrical remodeling, thus promoting arrhythmia, such as AF. POAF postoperative atrial fibrillation, CABG coronary artery bypass grafting, Kpr lysine propionylation, NADH nicotinamide adenine dinucleotide, ROS reactive oxygen species, I_kur_ ultra-rapid delayed rectifier K^+^ current, I_Na_ sodium current, I_Ca, L_ L-type calcium current. Some of the elements in this figure were created by Biorender.

Further, after transfection of ALDH6A1-K113Q in HEK293 cells, the level of ROS in the cells was significantly higher than that of transfection of ALDH6A1-K113 and ALDH6A1-K113R, which might be due to the increase of NADH in the cells (Fig. 5d, e). A study on ALDH6A1 as a potential biomarker of hepatocellular carcinoma showed that an increase in ALDH6A1 in ALDH6A1-O/E cells would cause a reduction in NAD^+^/NADH, which consequently disrupts mitochondrial membrane potential. The disruption in membrane potential would then lead to decreased NO and increased ROS levels in ALDH6A1-O/E cells. This is similar to the results from the present study^55^. Oxidative stress is mainly caused by excessive ROS and is associated with the development of cardiovascular diseases^56,57^. Excessive ROS targeted a variety of arrhythmogenic molecules, affecting ion channel alterations and electrical remodeling, thereby inducing AF^58,59^. The main channels include potassium^18^, sodium^17^, and calcium channels^19^. Kv1.5 channel, encoded by KCNA5 gene, is a specific ion channel of atrial myocytes and plays an important role in the process of action potential repolarization of atrial myocytes^60^. It was found that oxidative stress affected the post-translational modification of Kv1.5 channel protein and regulated the function of Kv1.5 channel^18^. Two independent studies have demonstrated that an increase in cellular NADH may lead to a decrease in sodium current (I_Na_) and was associated with arrhythmias^17,61^. This process was due to the fact that the increase in intracellular NADH activated ROS overproduction, which affected the sodium channel (Na_v_1.5) in the cell membrane^17^. Moreover, ROS also decreased the activity of L-type Ca^2+^ channel (LTCC) and Ca^2+^-ATPase (SERCA), and increased the activity of ryanodine receptor (RYR) and Na^+^/Ca^2+^ exchanger (NCX)^62,63^. This process inhibited sarcoplasmic reticulum (SR) Ca^2+^ reuptake during myocardial contraction and promoted intracellular Ca^2+^ leakage, affecting Ca^2+^ homeostasis, triggering electrical remodeling, and promoting arrhythmogenesis^64^. However, another study showed that the increase of cytosolic NADH/NAD^+^ level may regulate the release of SR Ca^2+^ in myocardial cells by inhibiting RyR channels and SR Ca^2+^ pumps^65^. In our study, propionylated ALDH6A1 caused the elevation of intracellular NADH as well as the accumulation of ROS. The latter may trigger oxidative stress and atrial electrical remodeling, and possibly promoting the development of POAF in CABG patients (Fig. 7). However, the specific mechanism of the ALDH6A1-NADH pathway regulating ion channels in atrial myocytes needs to be further studied. In addition, studies have shown that mitochondrial respiratory disorders are closely related to the occurrence of POAF^66,67^. We found that the ultrastructure of mitochondria in atrial tissue of POAF patients were different from the POSR patients even before the major procedure of CABG started, which is likely related to the obstruction of mitochondrial respiratory chain and an increase of NADH, triggering inflammation, oxidative stress, and affecting energy metabolism, thereby inducing POAF.

Many studies have shown that the decrease of LVEF may be used as an independent risk factor for POAF prediction^68–70^. We have recently analyzed 6229 patients who underwent CABG surgery at our hospital showing that lower LVEF is a risk factor for POAF^4^. The present study recruited 28 patients who underwent CABG, including 14 POAF patients and 14 POSR patients. Compared with POSR patients, POAF patients have significantly lower LVEF before CABG, which may lead to left ventricular systolic dysfunction, thus triggering cardiac structural remodeling, inflammatory reaction, and oxidative stress^71^. In addition, all the tissue samples were obtained during CPB intubation before operation. This indicates that the observed changes in protein propionylation level and mitochondrial ultrastructure reflect the pre-existing conditions before CABG, rather than the operation-related factors. These changes may induce oxidative stress and energy metabolism disorder, thereby promoting the occurrence of POAF.

### Limitations

There are also some limitations in our study. As usual in proteomics studies, the sample size was limited. However, the chosen proteins from the PTM proteomics were further validated in the new samples from a new cohort of patients and the validation was also performed at the cellular level. In addition, the regulation mechanism of the ALDH6A1-NADH pathway on ion channels needs to be further studied.

In conclusion, our study demonstrates that the occurrence of POAF in CABG patients is related to the changes of protein propionylation, especially to the increase in ALDH6A1-K113 propionylation. Increased Kpr level of ALDH6A1 promotes its enzyme activity, causing an increase in NADH and ROS. These effects may trigger oxidative stress, which promotes the occurrence of POAF after CABG. Thus, this study reveals that the ALDH6A1-NADH pathway is involved in the pathogenesis of POAF and provides new insights for the possible strategy of future therapeutic targets in order to improve clinical outcomes of CABG.

## Methods

### Clinical samples acquisition

All clinical tissue samples and clinical information in this study were approved by the Institutional Review Board of TEDA International Cardiovascular Disease Hospital and were performed with the informed consent of the patients.

We prospectively collected discarded right atrial appendage tissues from CABG patients during surgery and froze them. The final selection of the samples used for quantitative proteomics and propionylation quantitative proteomics was determined after the surgery. The samples were then allocated into the POSR or POAF group, depending on whether the patient was in SR or in AF within the postoperative days before discharge.

A total of 28 CABG patients (10 patients for the discovery group, 12 patients for the validation group, and 6 patients for transmission electron microscope) were included in our study by matching relevant clinical data such as age, gender, and race. All patients were in SR before surgery, of which 14 patients developed POAF, and 14 patients remained in POSR. Specific clinical information is shown in Supplementary Table 1.

The definition of POAF was consistent with our previous study^3^. Specifically, POAF patients had multiple episodes of AF lasting more than 30 s, recorded by continuous ECG monitoring or wireless ECG telemetry, from after CABG surgery alone to before discharge, and required intravenous amiodarone for anti-AF treatment. Exclusion criteria included multiple CBAG surgeries, CABG combined with valve replacement surgery, and other serious diseases.

**Fig. 8:**
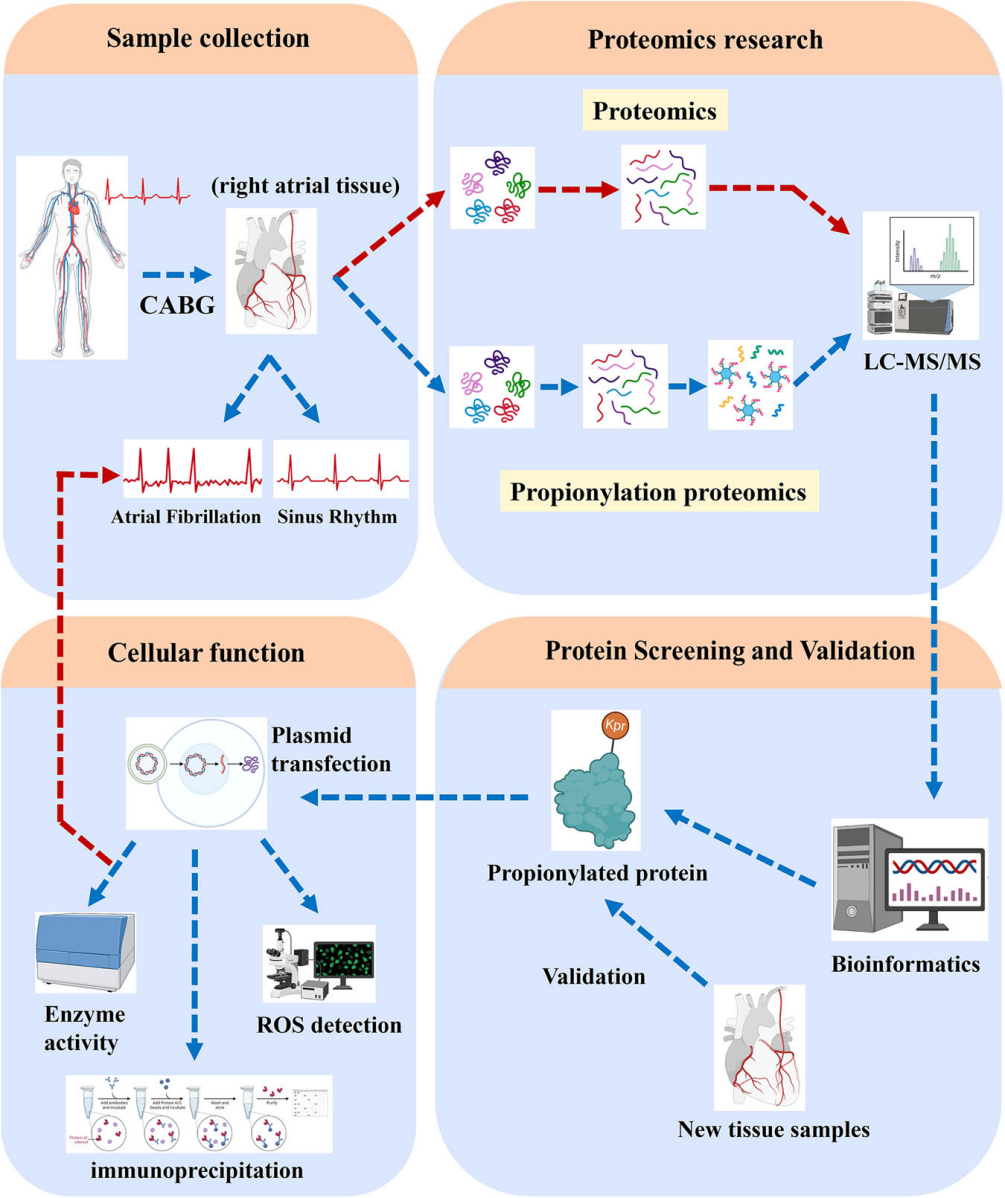
Study flow chart. The main research methods and technical routes in this study were illustrated in the flowchart. Some of the elements in this figure were created by Biorender.

The flow chart of this study is shown in Fig. 8.

### Proteomics and lysine propionylation proteomics analysis

The experiments of quantitative proteomics and propionylation proteomics were performed using the 4D Label-Free approach, with technical support provided by Jingjie PTM BioLab (Hangzhou) Co., Ltd. The right atrial appendage tissues (POSR, n = 5; POAF, n = 5) were grinded in liquid nitrogen, and then subjected to protein extraction, trypsin digestion, affinity enrichment (PTM-202, PTM BioLab, used in propionylation proteomics analysis), and eluted to detect peptides using liquid chromatography-mass spectrometry (LC-MS/MS). Finally, bioinformatics tools were used to analyze the proteomics data, including proteomics and propionylation proteomics association analysis, modification sites motif analysis, GO enrichment analysis, KEGG pathway enrichment analysis, protein domain enrichment analysis, and protein interaction analysis.

The data of protein propionylation were normalized with the conventional quantitative proteomics data from the same samples. The relative ratio of AF/SR > 1.5 or < 1/1.5, and the *P* value < 0.05, were considered to be significantly increased or decreased in protein propionylation level, respectively.

### Immunoprecipitation (IP) and western blot (WB) analysis

We verified the propionylated level of expressed ALDH6A1 in right atrial appendage tissues from patients with POSR and POAF using a combination of immunoprecipitation (IP) and immunoblotting techniques. Approximately 50 mg of tissues were lysed by cell lysis buffer for WB and IP (1% PMSF, 1% NAM, 0.1% TSA), and the protein concentration were determined by BCA kit. For IP, ALDH6A1 monoclonal antibody (F-10, mouse, SANTA) was added to the protein lysates and incubated overnight at 4 °C, Protein A + G Agarose (P2055, Beyotime) was added and incubated at room temperature for 3 h, and then washed and resuspended for SDS-PAGE electrophoresis. For WB, 10% SDS-PAGE was used for electrophoresis. After blocking, the membrane was incubated overnight with the primary antibody (anti-propionyllysine rabbit pAb, PTM-201, PTM BioLab) at 4 °C, and then incubated in mouse anti-rabbit IgG light-chain specific mAb (HRP conjugate, 93702, Cell Signaling Technology) for 1 h at room temperature. The GAPDH was chosen as an internal reference (10494-1-AP, rabbit, Proteintech). Band signals were visualized and quantified by G:BOX Chemi XR5 gel documentation system (Syngene). Then, the distinction of propionylated ALDH6A1 protein between POSR and POAF was further validated by ROC curve.

The expression of protein was verified in cells in the same way as described above.

### Construction of mutant plasmids

The *Human ALDH6A1* cDNA ORF clone expression plasmid (pCMV3-Flag-*ALDH6A1*, HG22618-NF) and negative control vector (pCMV3-Flag, CV016) were purchased from Sino Biological. The lysine located at position 113 of pCMV3-ALDH6A1-K113 was mutated into glutamine (pCMV3-ALDH6A1-K113Q) or arginine (pCMV3-ALDH6A1-K113R) using QuickMutation^TM^ Site-Directed Mutagenesis Kit (D0206S, Beyotime) to simulate increased or decreased levels of propionylation, respectively. The mutant primer sequences are shown in Supplementary Table 2. The constructed plasmids were separately transferred into *E.coli* DH5α chemically competent cells for culture, and fresh plasmids were extracted for subsequent transfection.

### Cell lines and culture

Adherent HEK293 cells were cultured in DMEM (11965084, Gibco) containing 10% Fetal Bovine Serum (FSP500, Excell) as well as 1% Penicillin-Streptomycin Liquid (P1400, Solarbio). One day before transfection, 6 × 10^5^ cells were spread into 6-well plates. When the cell density reached about 60–70%, 2.5 μg of the vectors pCMV3-Flag and pCMV3-ALDH6A1-Flag (K113, K113Q, and K113R) were transfected into the cells using Lipo293^TM^ reagent (C0521, Beyotime), respectively. The subsequent assays were carried out 24–48 h later. Among them, pCMV3-Flag was used as an empty control.

### ALDH6A1 enzyme activity assay

ALDH6A1 has the ability to reduce NAD^+^ to NADH during catalytic oxidative decarboxylation. The enzyme activity of ALDH6A1 is indirectly reacted by detecting the relative product formation of NADH in cells. After transfected pCMV3-Flag and pCMV3-ALDH6A1-Flag (K113, K113Q and K113R) in HEK293 cells for 30 h, the level of NADH was detected by the (NAD^+^/NADH Assay Kit with WST-8 (S0175, Beyotime) for each group, respectively. The value of pCMV3-Flag was subtracted from the value of each group of K113, K113Q, and K113R to remove the levels of NADH in the background. Normalization was then performed by quantifying Flag-ALDH6A1 protein for each group.

### Detection of intracellular ROS level

The ROS levels in transfected HEK293 cells were assayed using Reactive Oxygen Species Assay Kit (S0033S, Beyotime)^72,73^. DCFH-DA is non-fluorescent and can freely cross the cell membrane. After entering the cell, DCFH-DA is hydrolyzed to form DCFH. The ROS can oxidize the non-fluorescent DCFH to produce green fluorescent DCF. The intracellular ROS levels were detected by observing the fluorescence of DCF by fluorescence microscopy.

### Simulation of binding of ALDH6A1 and NAD^+^

The original PDB structure of *Human* ALDH6A1 protein (Q02252) was downloaded from UniProt (https://alphafold.ebi.ac.uk/entry/Q02252). The structure of NAD^+^ was obtained from Chemspider (https://www.chemspider.com). A propionyl was added to the side chain of ALDHA61-K113 using PyMOL software to simulate an increased level of propionylation. Molecular docking of ALDH6A1 (with or without Kpr) with NAD^+^ was performed using AutoDock vina software. The analysis was performed by PyMOL visualization to observe the changes in the binding conformation and binding energy to determine the effect of Kpr modification on ALDH6A1 enzyme activity.

### Transmission electron microscope (TEM) analysis

The right atrial appendage tissues discarded during CABG surgery were collected from three POSR patients and three POAF patients and fixed in 2.5% glutaraldehyde for 2–4 h. The samples were rinsed with phosphoric acid and fixed in 1% osmium acid at 4 °C for 2 h, and then dehydrated with an ethanol gradient. After resin embedding, the slices were sliced with a Leica UC7 ultrathin slicer, and the slices were double-stained with uranyl acetate and lead citrate. The ultrastructure of tissues was observed with a JEM1400 transmission electron microscope.

### Statistics and reproducibility

All data are expressed as Mean ± SD. The Shapiro-Wilk test and Kolmogorov-Smirnov test are used to test the normality of the distribution. Student’s t-test and ANOVA were used separately to compare the differences between two groups or more groups. All data analyses were performed using GraphPad Prism 8 software, and *P* < 0.05 was considered a significant difference.

### Reporting summary

Further information on research design is available in the Nature Portfolio Reporting Summary linked to this article.

## Supplementary information

Supplementary Information

Description of Additional Supplementary Files

Supplementary Data

Reporting Summary

## Data Availability

The mass spectrometry proteomics data and propionylation proteomics data have been deposited to the ProteomeXchange Consortium via the PRIDE partner repository under accession numbers PXD059685 and PXD059607. Additionally, all source data underlying the graphs and charts presented in the main figures are provided in Supplementary Data 1.
