# Supplementary material for: Atlas of lysine propionylation of human right atrium and ALDH6A1-NADH pathway in new-onset atrial fibrillation after coronary surgery
Source: Commun Biol. 2025 Jun 4;8:858. doi: 10.1038/s42003-025-08264-9 (PMC12137918; doi:10.1038/s42003-025-08264-9)
Supplement: Supplementary file 1 — Supplementary Information [file 42003_2025_8264_MOESM1_ESM.pdf]

Supplementary Information

Supplementary Table 1 Baseline characteristics of patients with POSR and POAF after CABG

Variable	POSR (n = 14)	POAF (n = 14)	*P* value
Age (years)	68.00 ± 5.63	69.21 ± 5.55	0.570
Male sex	8 (57.14)	8 (57.14)	> 0.999
BMI	25.07 ± 3.02	25.87 ± 3.66	0.534
Smoking	8 (57.14)	5 (35.71)	0.256
Hypertension	11 (78.75)	11 (78.75)	> 0.999
Fasting blood glucose (mmol/L)	5.76 ± 1.60	6.54 ± 2.14	0.285
CREA (mmol/L)	64.79 ± 15.76	66.76 ± 17.93	0.756
TCHOL (mmol/L)	4.08 ± 0.83	4.24 ± 1.09	0.671
LDL-C (mmol/L)	2.47 ± 0.72	2.47 ± 0.87	0.998
LVEF (%)	65.71 ± 4.41	57.29 ± 9.62	0.006
LVDd (mm)	46.07 ± 3.93	46.71 ± 5.01	0.709
LADs (mm)	36.21 ± 4.30	35.29 ± 4.73	0.591

These values are expressed as the mean ± SD. N = 28. POSR, Postoperative sinus rhythm; POAF, Postoperative atrial fibrillation; CABG, Coronary artery bypass grafting; BMI, Body massive index; CREA, Creatinine; TCHOL, Total cholesterol; LDL-C, Low-density lipoprotein cholesterol; LVEF, Left ventricular ejection fraction; LVDd, Left ventricular diastolic diameter; LADs, Left atrial end systolic diameter.

Supplementary Table 2 Primers sequences for targeted mutagenesis

ALDH6A1 mutants	Primer sequences (5’-3’)
ALDH6A1-K113	F: GCTCCGCTATCAACAACTTATTAAAGAAAAC TTGAAAGAAATTGC<br>R: GCAATTTCTTTCAAGTTTTCTTTAATAAGTTG TTGATAGCGGAGC
ALDH6A1-K113Q	F: GCTCCGCTATCAACAACTTATTCAAGAAAAC TTGAAAGAAATTGC<br>R: GCAATTTCTTTCAAGTTTTCTTGAATAAGTTG TTGATAGCGGAGC
ALDH6A1-K113R	F: GCTCCGCTATCAACAACTTATTAGAGAAAAC TTGAAAGAAATTGC<br>R: GCAATTTCTTTCAAGTTTTCTCTAATAAGTTG TTGATAGCGGAGC

Supplementary Figure S1

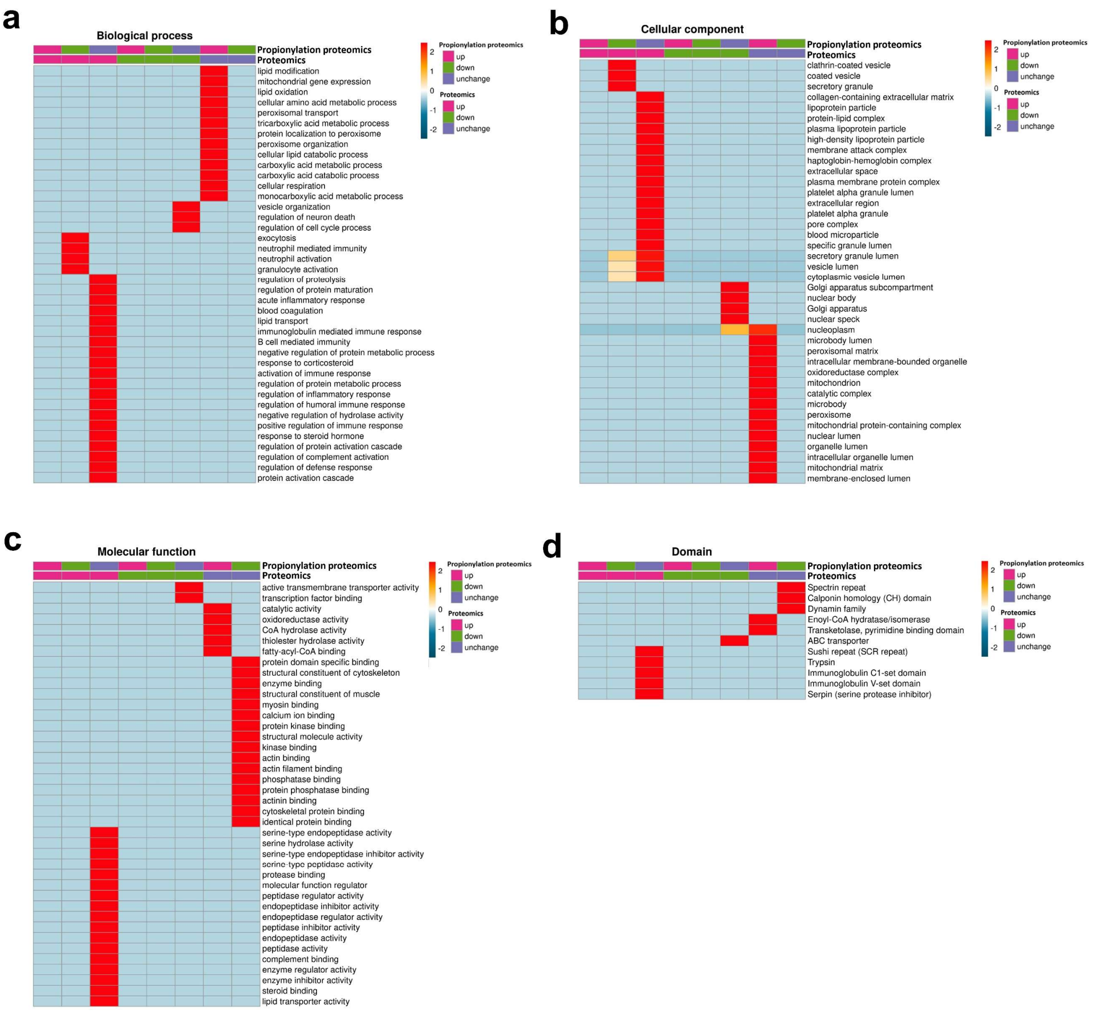

Supplementary Fig. S1: Bioinformatics analysis of the correlation between Proteomics and propionylated Proteomics of right atrial appendage in patients with POSR and POAF.

a, b, c Heat map of functional classification differences between the two omics of differently classified proteins on GO analysis (including biological process, cellular component and molecular function). **d** Heat map of functional enrichment differences between the two omics of differently classified proteins on domain. The redder the color, the more significant the enrichment.

Supplementary Figure S2

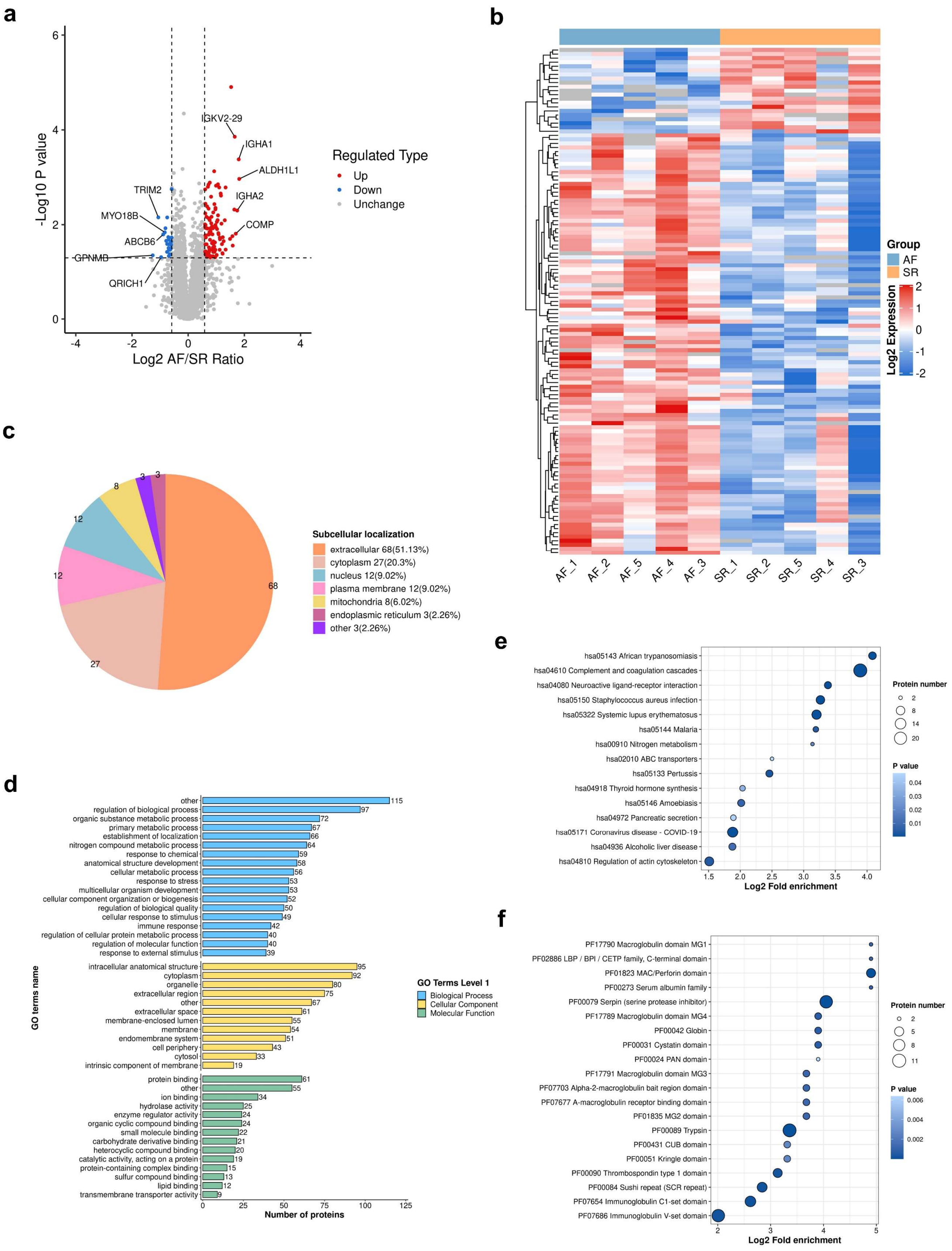

Supplementary Figure S2 Continued

g

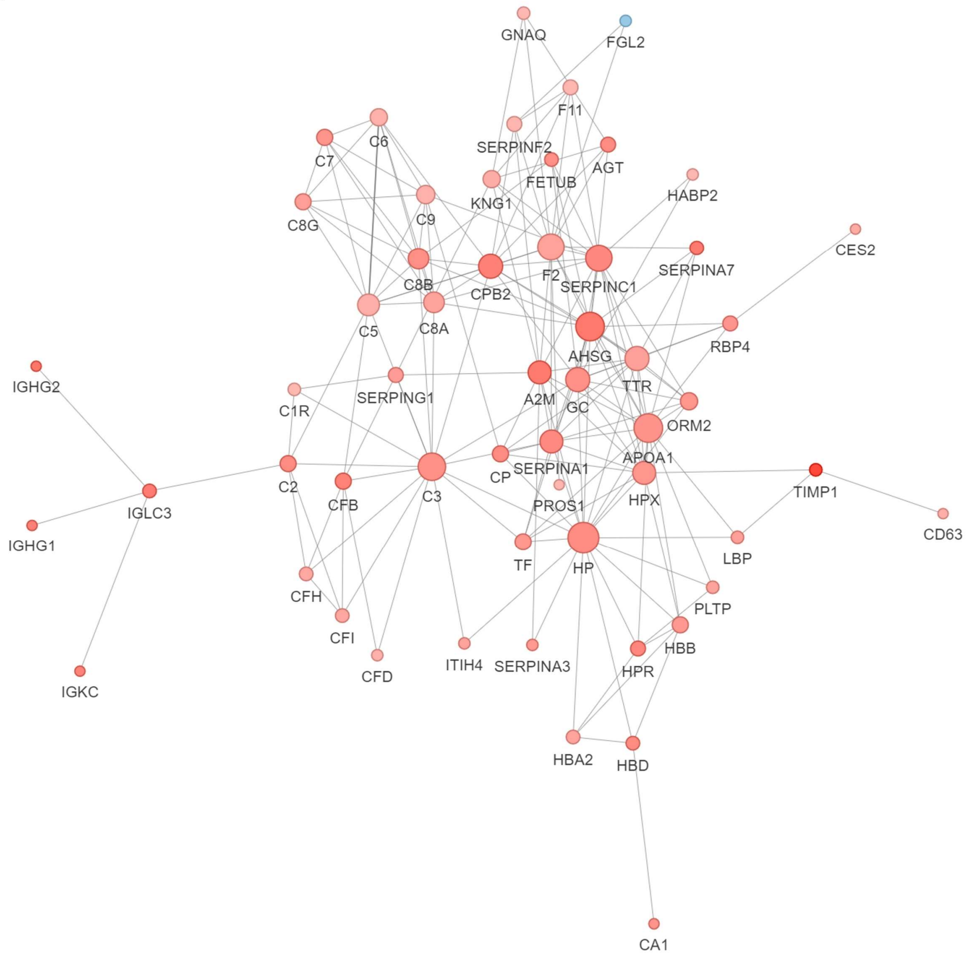

Supplementary Fig. S2: Proteomics analysis in intraoperative right atrial appendage tissues from patients with POSR and POAF.

a The volcano plot of differential proteins. The red points indicated significant up-regulation, and the blue points indicated significant down-regulation. The information on the differentially modified sites for Top5 up- and down-regulation were also marked in the figure, respectively. **b** The heat map of differential proteins. Red represented high expression and blue represented low expression. **c** The number of differentially protein in different subcellular structural types. **d** GO enrichment analysis of differentially proteins based on biological process, cellular component and molecular function. **e** KEGG pathway analysis of differentially proteins. **f** Protein domain enrichment analysis of differentially proteins. **g** Diagram of the tightest 50 differential protein-protein interaction network.

Supplementary Figure S3

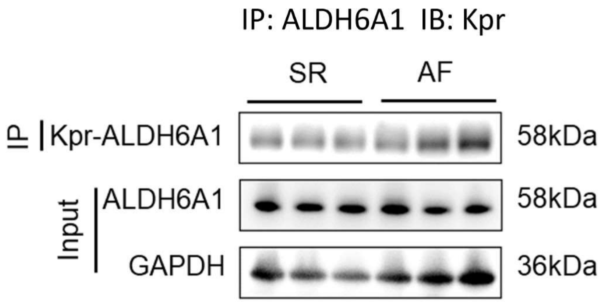

Supplementary Fig. S3: Up-regulated propionylation expression of ALDH6A1 in right atrial appendage tissue of POAF.

Validation of ALDH6A1 propionylated expression in POSR and POAF right atrial appendage tissues by IP and WB (n=3 per group).

Supplementary Figure S4

Fig. 4d

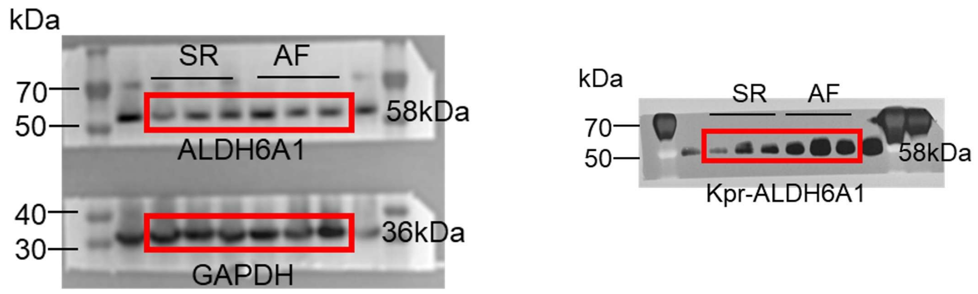

Fig. 5b

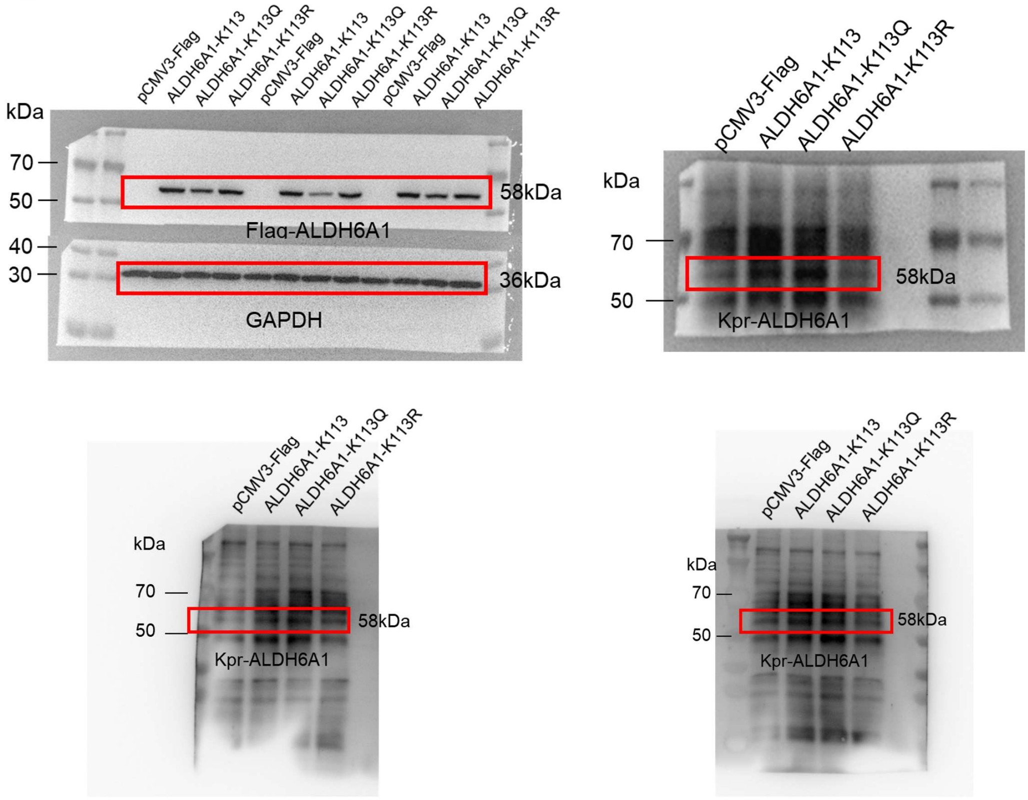

Supplementary Fig. 3

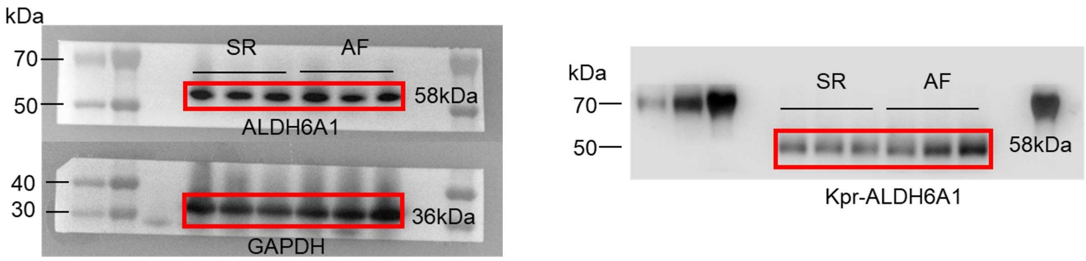

Supplementary Fig. S4: Uncropped and unedited blots images.
