# Supplementary material for: Atlas of lysine propionylation of human right atrium and ALDH6A1-NADH pathway in new-onset atrial fibrillation after coronary surgery
Source: Commun Biol. 2025 Jun 4;8:858. doi: 10.1038/s42003-025-08264-9 (PMC12137918; doi:10.1038/s42003-025-08264-9)
Supplement: Supplementary file 4 — Reporting Summary [file 42003_2025_8264_MOESM4_ESM.pdf]

nature portfolio

Corresponding author(s): Guo-Wei He

Last updated by author(s): May 9, 2025

Reporting Summary

Nature Portfolio wishes to improve the reproducibility of the work that we publish. This form provides structure for consistency and transparency in reporting. For further information on Nature Portfolio policies, see our Editorial Policies and the Editorial Policy Checklist.

Statistics

For all statistical analyses, confirm that the following items are present in the figure legend, table legend, main text, or Methods section.

n/a	Confirmed	
☐	☒	The exact sample size (*n*) for each experimental group/condition, given as a discrete number and unit of measurement
☐	☒	A statement on whether measurements were taken from distinct samples or whether the same sample was measured repeatedly
☐	☒	The statistical test(s) used AND whether they are one- or two-sided<br>*Only common tests should be described solely by name; describe more complex techniques in the Methods section.*
☐	☒	A description of all covariates tested
☐	☒	A description of any assumptions or corrections, such as tests of normality and adjustment for multiple comparisons
☐	☒	A full description of the statistical parameters including central tendency (e.g. means) or other basic estimates (e.g. regression coefficient) AND variation (e.g. standard deviation) or associated estimates of uncertainty (e.g. confidence intervals)
☒	☐	For null hypothesis testing, the test statistic (e.g. *F*, *t*, *r*) with confidence intervals, effect sizes, degrees of freedom and *P* value noted<br>*Give P values as exact values whenever suitable.*
☒	☐	For Bayesian analysis, information on the choice of priors and Markov chain Monte Carlo settings
☒	☐	For hierarchical and complex designs, identification of the appropriate level for tests and full reporting of outcomes
☒	☐	Estimates of effect sizes (e.g. Cohen's *d*, Pearson's *r*), indicating how they were calculated

Our web collection on statistics for biologists contains articles on many of the points above.

Software and code

Policy information about availability of computer code

Data collection

The data of patient's clinical samples were obtained from TEDA International Cardiovascular Hospital in Tianjin, China.
The proteomics and propionylated proteomics data were obtained from the 4D label free technique of Jingjie PTM Biolab Co. Ltd. (Hangzhou, China).
Western blots were acquired on G:BOX Chemi XR5 gel documentation system (Syngene).
The original PDB structure of protein was downloaded from Uniport.
The structure of small molecular was obtained from Chemspider.

Data analysis

All software used to analyze the data is commercial or open-source.
LC-MS/MS data were analyzed with MaxQuant search engine (v.1.6.15.0).
Pathway analysis were performed with eggnog-mapper (v2.1.6), diamond (v2.0.11.149) and PfamScan (v1.6).
Statistics were analyzed using GraphPad Prism software 8.0.
Image data analysis was performed using Image J software.
Molecular docking was performed using AutoDock vina software.
The visualization analysis was performed by PyMOL 2.5.2.
The ultrastructure of tissues was observed with a JEM1400 transmission electron microscope.
Statistics were analyzed using GraphPad Prism software 8.0.

For manuscripts utilizing custom algorithms or software that are central to the research but not yet described in published literature, software must be made available to editors and reviewers. We strongly encourage code deposition in a community repository (e.g. GitHub). See the Nature Portfolio guidelines for submitting code & software for further information.

Data

Policy information about availability of data

All manuscripts must include a data availability statement. This statement should provide the following information, where applicable:

- Accession codes, unique identifiers, or web links for publicly available datasets
- A description of any restrictions on data availability
- For clinical datasets or third party data, please ensure that the statement adheres to our policy

The data supporting the findings of this study are available within the paper and its Supplementary Information. The mass spectrometry proteomics data have been deposited to the ProteomeXchange Consortium via the PRIDE partner repository with the dataset identifier PXD059685. Token: Ppi5nLM4VDeB. The mass spectrometry propionylated proteomics data have been deposited to the ProteomeXchange Consortium via the PRIDE partner repository with the dataset identifier PXD059607. Token: Tw5c4vy8EEjg

Research involving human participants, their data, or biological material

Policy information about studies with human participants or human data. See also policy information about sex, gender (identity/presentation), and sexual orientation and race, ethnicity and racism.

Reporting on sex and gender	Information on sex has been collected and reported in the manuscript (Supplementary Table 1). Diabetes, hypertension,obesity, sleep-related breathing disorders and heart failure increase the risk of AF development with many of these risk factors being more prevalent in male, thus the higher proportion of males in our clinical cohort. For this reason, sex-based analysis was not possible due to low power. Informed consent was obtained for all patients.
Reporting on race, ethnicity, or other socially relevant groupings	Information on race, ethnicity, or other social groupings was not included in our data or analysis.
Population characteristics	Population characteristics are included in Extended Data Table 1.
Recruitment	The patients in this study were recruited by TEDA International Cardiovascular Hospital in Tianjin, China. Inclusion criteria were preoperative sinus rhythm in patients who were to undergo CABG alone. Exclusion criteria were preoperative arrhythmia, repeat CABG, CABG combined with heart valve surgery, severe cerebral, pulmonary, or renal disease, age >80, and severe metabolic disease.The patients were classified into atrial fibrillation (AF) and non-atrial fibrillation (sinus rhythm) groups according to the presence or absence of AF at the time of discharge from the hospital after CABG. The two groups were matched as closely as possible for clinical information such as age, gender, and race.
Ethics oversight	All clinical tissue samples and clinical information in this study were approved by the Institutional Review Board of TEDA International Cardiovascular Disease Hospital and were performed with the informed consent of the patients.

Note that full information on the approval of the study protocol must also be provided in the manuscript.

Field-specific reporting

Please select the one below that is the best fit for your research. If you are not sure, read the appropriate sections before making your selection.

☒ Life sciences ☐ Behavioural & social sciences ☐ Ecological, evolutionary & environmental sciences

For a reference copy of the document with all sections, see nature.com/documents/nr-reporting-summary-flat.pdf

Life sciences study design

All studies must disclose on these points even when the disclosure is negative.

Sample size	Our cohort included 28 CABG patients (n=14 POAF patients, n=14 POSR patients). The proteomics and propionylation proteomis cohort included n=5 POAF patients and n=5 POSR patients. The protein validation cohort included n=6 POAF patients and n=6 POSR patients. Transmission Electron Microscope cohort included n=3 POAF patients and n=3 POSR patients. POAF was defined as multiple AF lasting >30 seconds, recorded by electrocardiogram monitor or continuous wireless rhythmic monitoring, began immediately after surgery or later before discharge, requiring anti-AF treatment (usually intravenous amiodarone).
Data exclusions	Exclusion criteria were: preoperative arrhythmia, repeat CABG surgery, CABG combined with heart valve surgery, combination of severe brain, lung, and kidney disease, age >80, and severe metabolic disease.
Replication	All data were reproducible, and successfully replicated. All replicates for each type of experiment are indicated in the manuscript. A minimum of three biological replicates were used for most experiments.
Randomization	The atrial tissues of POAF patients after CABG were randomly distributed in different experimental groups, and the atrial tissues of POSR patients after CABG were randomly distributed in different control groups.

Blinding	The investigators responsible for acquiring data were blinded to the experimental groups, Data analysis was also performed blinded whenever possible.

Reporting for specific materials, systems and methods

We require information from authors about some types of materials, experimental systems and methods used in many studies. Here, indicate whether each material, system or method listed is relevant to your study. If you are not sure if a list item applies to your research, read the appropriate section before selecting a response.

Materials & experimental systems

n/a	Involved in the study	
☐	☒	Antibodies
☐	☒	Eukaryotic cell lines
☒	☐	Palaeontology and archaeology
☒	☐	Animals and other organisms
☒	☐	Clinical data
☒	☐	Dual use research of concern
☒	☐	Plants

Methods

n/a	Involved in the study	
☒	☐	ChIP-seq
☒	☐	Flow cytometry
☒	☐	MRI-based neuroimaging

Antibodies

Antibodies used	All antibody details are included in methods and also listed below:<br>ALDH6A1 monoclonal antibody (sc-365160, 1:500, Santa Cruz);<br>anti-propionyllysine rabbit pAb (PTM-201, 1:1000, PTM BioLab);<br>GAPDH (10494-1-AP, 1:2000, Proteintech);<br>mouse anti-rabbit IgG light-chain specific mAb (93702, 1:2000, Cell Signaling);<br>Anti-mouse IgG, HRP-linked Antibody (7076S, 1:2000, Cell Signaling);<br>Anti-rabbit IgG, HRP-linked Antibody (7074S, 1:2000, Cell Signaling).
Validation	All antibodies used in this study are commercially obtained and were validated commercial suppliers.

Eukaryotic cell lines

Policy information about cell lines and Sex and Gender in Research

Cell line source(s)	HEK293 cell line were purchased from ATCC.
Authentication	Cell line was authenticated by the vendor.
Mycoplasma contamination	negative
Commonly misidentified lines (See ICLAC register)	No commonly misidentified cell lines were used in this study.

Plants

Seed stocks	n/a
Novel plant genotypes	n/a
Authentication	n/a
